# A Trafficking-Deficient Mutant of KCC3 Reveals Dominant-Negative Effects on K–Cl Cotransport Function

**DOI:** 10.1371/journal.pone.0061112

**Published:** 2013-04-04

**Authors:** Jinlong Ding, José Ponce-Coria, Eric Delpire

**Affiliations:** 1 Department of Anesthesiology, Vanderbilt University School of Medicine, Nashville, Tennessee, United States of America; 2 Molecular Physiology and Biophysics Graduate Program Vanderbilt University School of Medicine, Nashville, Tennessee, United States of America; University of Toronto, Canada

## Abstract

The K–Cl cotransporter (KCC) functions in maintaining chloride and volume homeostasis in a variety of cells. In the process of cloning the mouse KCC3 cDNA, we came across a cloning mutation (E289G) that rendered the cotransporter inactive in functional assays in *Xenopus laevis* oocytes. Through biochemical studies, we demonstrate that the mutant E289G cotransporter is glycosylation-deficient, does not move beyond the endoplasmic reticulum or the early Golgi, and thus fails to reach the plasma membrane. We establish through co-immunoprecipitation experiments that both wild-type and mutant KCC3 with KCC2 results in the formation of hetero-dimers. We further demonstrate that formation of these hetero-dimers prevents the proper trafficking of the cotransporter to the plasma membrane, resulting in a significant decrease in cotransporter function. This effect is due to interaction between the K–Cl cotransporter isoforms, as this was not observed when KCC3-E289G was co-expressed with NKCC1. Our studies also reveal that the glutamic acid residue is essential to K–Cl cotransporter function, as the corresponding mutation in KCC2 also leads to an absence of function. Interestingly, mutation of this conserved glutamic acid residue in the Na^+^-dependent cation-chloride cotransporters had no effect on NKCC1 function in isosmotic conditions, but diminished cotransporter activity under hypertonicity. Together, our data show that the glutamic acid residue (E289) is essential for proper trafficking and function of KCCs and that expression of a non-functional but full-length K–Cl cotransporter might results in dominant-negative effects on other K–Cl cotransporters.

## Introduction

In mammals, electroneutral and Na^+^-independent K–Cl cotransport is mediated by 4 distinct genes: SLC12A4-A7 (for review, see [1]). The products of these genes (KCC1-KCC4) fulfill a variety of physiological roles which include cell volume maintenance and regulation [2], Cl^−^ homeostasis [3], [4], [5], epithelial transport [6], control of migration, proliferation, and invasiveness [7]. K–Cl cotransporters are regulated phosphatases [8], [9] and the WNK-SPAK/OSR1 phosphorylation cascade [10], [11], [12], [13], [14], [15].

Mutations in SLC12A6, the gene which encodes for the K–Cl cotransporter-3 (KCC3), results in a rare autosomal recessive neurological disorder known as Hereditary Motor and Sensory Neuropathy/Agenesis of the Corpus Callosum (HSMN/ACC) (OMIM 218000; [16], [17]). The pathological hallmarks of this syndrome, with high prevalence in the French-Canadian population of Quebec, are a peripheral neuropathy which is often associated with variable agenesis of the corpus callosum, areflexia, mental retardation, and psychosis [18], [19]. KCC3-deficient mice exhibit not only the early onset and severe locomotor deficits similar to the crippling human peripheral neuropathy disorder [16], but also high blood pressure [20], [21], age-related deafness, and renal dysfunction [17]. At the ultrastructural level, KCC3-deficient mice exhibit axonal and peri-axonal swelling indicating both neuronal and Schwann cell defects [22]. A recent study that used a synapsin 1-CRE mouse to drive deletion of neuronal KCC3 expression reproduced the neuropathy phenotype observed in the KCC3 knockout mouse [23].

Injection of KCC3-T813X, the prevalent mutation observed in the French-Canadian population, in *Xenopus laevis* oocytes demonstrated expression of a glycosylated protein of a smaller molecular size at or near the oocyte plasma membrane similar to wild-type KCC3 [16]. In contrast, a novel and more distal HMSN/ACC truncating mutant (KCC3-R1134X) failed to traffic properly to the plasma membrane [24].

Several studies have reported expression of more than one K–Cl cotransporter isoform in cells and tissues, including red blood cells [25]; glial cells [26]; vascular smooth muscle cells [27]; and suprachiasmatic neurons [28]. In fact, most large CNS neurons such as cortical or hippocampal pyramidal cells express both KCC2 and KCC3 [29], and disruption of either cotransporter elicits shifts in the GABA reversal potential [17]. The fact that cells express multiple KCC isoforms suggests the intriguing possibility that these cotransporters interact with one another, a view supported by an early evidence of isoform hetero-dimerization [30].

In this study, we take advantage of a full-length KCC3-E289G mutant that maintains the entire open reading frame, but renders the cotransporter functionally inactive to assess interaction, trafficking, and function of co-expressed K-Cl cotransporters. While this mutation is not found in nature, it still provides important information on a specific residue of the K–Cl cotransporter and constitutes a very useful tool to study its trafficking and hetero-oligomerization. We provide evidence that K–Cl cotransporter isoforms interact in *Xenopus laevis* oocytes and affect each other's function. We demonstrate that the mutant KCC3-E289G protein resides in the endoplasmic reticulum (ER), is not properly glycosylated, and does not traffic to the plasma membrane. Furthermore, we show that the E289G mutant also prevents wild-type KCC3 as well as another K–Cl cotransporter isoform to traffic to the plasma membrane.

## Methods

### Cloning of mouse KCC3 cDNA

The entire open reading frame of the mouse KCC3a was constructed by ligating into Bluescript (pBSK+) several PCR fragments obtained from David B. Mount (Vanderbilt University). The clone was sequenced and moved into the oocyte expression vector pBF. As the cDNA failed to demonstrate K^+^ transport, the cDNA was re-sequenced and an adenine to guanine substitution at nucleotide position 866, leading to mutation of glutamic acid residue 289 into a glycine was identified. After the mutation was corrected using QuikChange mutagenesis, the clone was re-sequenced and re-tested for functionality.

#### Isolation of *Xenopus laevis* oocytes

All procedures performed with frogs were approved by the Vanderbilt University Institutional Animal Care and Use Committee. Stages V and VI *Xenopus laevis* oocytes were isolated from 16 different frogs as previously described [31] and were maintained at 16°C in modified L15 medium (Leibovitz's L15 solution diluted with water to a final osmolarity of 195–200 mOsM, supplemented with 10 mM HEPES and 44 µg of gentamicin sulfate). The next day, oocytes were injected with 50 nl of water containing 2–15 ng of cotransporter cRNA (concentrations are indicated in specific experiments) and oocytes are incubated for 3 days prior to use.

#### K^+^ uptakes in *Xenopus laevis* oocytes

Groups of 20–25 oocytes were washed once with 3 ml isosmotic saline (96 mM NaCl, 4 mM KCl, 2 mM CaCl_2_ 1 mM MgCl_2_, 5 mM HEPES buffered to pH 7.4, 200 mOsM) and pre-incubated for 15 min in 1 ml Na^+^ free isosmotic or hyposmotic saline containing 1 mM ouabain. The solution was then aspirated and replaced with 1 ml Na^+^ free isosmotic or hyposmotic flux solution containing 5 µCi ^86^Rb. Two 5 µl aliquots of flux solution were sampled at the beginning of each ^86^Rb uptake period and used as standards. After 1 h uptake at room temperature, the radioactive solution was aspirated and the oocytes were washed 4 times with 3 ml ice-cold Na^+^ free isosmotic or hyposmotic solution. Single oocytes were transferred into glass vials, lysed for 1 h with 200 µl 0.25N NaOH, neutralized with 100 µl glacial acetic acid, and ^86^Rb tracer activity was measured by β-scintillation counting. KCC flux is expressed in nmoles K^+^/oocyte/h.

#### Immunoprecipitation

Stage V–VI *Xenopus laevis* oocytes were microinjected with 15 ng each mKCC3 and/or rKCC2 cRNAs and incubated for 3 days at 16°C. Oocytes were then homogenized by passing them through a pipet tip (50 µl/oocyte) in 50 mM HEPES supplemented with Complete™ Protease Inhibitor Cocktail Tablet, EDTA-free (Roche). Homogenates were then centrifuged at 15,000 x g for 1 min and supernatants were saved for protein assay (Bradford, BioRad). An equal amount of protein was added to the HEPES buffer to a final volume of 1 ml. Immunoprecipitation was achieved by adding 10 µl of anti-GFP (IgG negative control), anti-KCC3, or anti-KCC2 antibody to the homogenate sample under gentle rotation overnight at 4°C. Then, 30 µl of pre-washed Protein A sepharose (Santa Cruz Biotechnology, Santa Cruz, CA) was added to each homogenate and incubated for 2 h at 4°C. The sepharose beads or expected immunoprecipitates were washed three times with 1 ml of lysis buffer (centrifugation at 4 °C, 2000 rpm, 2 min). The immunoprecipitates were re-suspended in 75 µl of loading buffer that contained 8% β-mercaptoethanol, heated at 75°C for 15 min, and subjected to SDS-PAGE.

#### Western Blot Analysis

Protein samples were denatured in SDS-PAGE loading buffer at 75°C for 15 min and separated on a 6%, 7.5% or 9% SDS-polyacrylamide gels. The separated proteins were electroblotted onto polyvinylidine fluoride membranes through a semi-dry process, and membranes were incubated for 2 h at room temperature in blocking solution (5% nonfat milk in Tris-buffered saline with 0.5% Tween 20). Incubation of primary antibody was performed overnight at 4 °C. The dilutions used for the antibodies were: KCC3 1:250, KCC2 1:250, PDI-ER marker 1∶1000, GAPDH-cytosol marker 1∶1000. Membranes were washed in TBST for 3 h, incubated with their corresponding horseradish peroxidase-conjugated secondary antibody in blocking solution (1∶5000) for 1 h at RT, and washed again for 2 h in TBST. Protein bands were visualized by chemiluminescence (ECL Plus, Amersham Biosciences).

#### Cell surface expression in *Xenopus laevis* oocytes

Isolation of plasma membrane bound proteins was done following [32]. Briefly, 3 days after microinjection of cRNA, 40–60 oocytes were rinsed three times in modified MBSS (80 mM NaCl, 20 mM MOPS pH 6.0) and incubated for 10 min at room temperature with modified MBSS supplemented with 0.005% subtilisin A (Sigma, St. Louis, MO) under gentle agitation to promote vitelline membrane digestion. Membrane polymerization was performed at 4°C under mild agitation by first incubating the oocytes with modified MBSS with 1% ludox colloidal silica (Sigma), and then with modified MBSS with 0.1% polyacrylic acid (Sigma). Between each incubation period, oocytes were rinsed three times in modified MBSS. The oocytes were then homogenized with 0.5 ml of cold HbA (5 mM MgCl_2_, 5 mM NaH_2_PO_4_, 1 mM EDTA, 80 mM sucrose, and 20 mM Tris pH 7.4). This homogenization was carried out manually by passing the oocytes through a 200 ul pipette tip. The homogenates were washed with 1.5 ml with HbA and centrifuged at 16 g for 30 sec at 4°C. The pellets were resuspended and subjected to a series of centrifugations: 16 g, 25 g and 35 g for 30 sec and max speed for 20 min. The pellets of purified plasma membranes were resuspended in 45 µl of HbA and frozen until use.

#### Cell culture and Transfection

HEK 293FT (Invitrogen, Carlsbad, CA) cells were maintained and routinely passaged in DMEM-F12 supplemented with 10% fetal bovine serum and 1% Penicillin/Streptomycin (Invitrogen) at 37°C under 95% air, 5% CO_2_. For transfection, cells were trypsinized and plated at 30% density the day prior to transfection. The cDNAs were then transfected into the cells using FuGENE 6 (Roche Applied Science) at a 3∶1 ratio (DNA:transfection reagent). Transfected cells were incubated at 37°C under 95% air, 5% CO_2_ for 48 h prior to use.

#### HEK 293FT sub-cellular fractionation

The method was adapted from [33]. HEK 293FT cells grown in 10-cm dishes were transfected with 16 ug of KCC3-pIRES _puro2 or KCC2-pIRES_puro2 and 48 h later, the culture medium was removed and cells were detached with 500 µL of Trypsin-EDTA (0.05%, Invitrogen). Cells were resuspended in 10 ml of complete culture medium, centrifuged at 100 x g at 4°C for 2 min. Next, the supernantant was aspirated and the cells were washed by gentle pipetting in 10 ml of Hank's Balanced Salt Solution. Cell suspension was centrifuged again at 100 x g at RT to pellet the cells. Supernatant was removed and the cell pellet was resuspended by gently adding 1 ml of ice cold buffer 1 (25 µg/ml digitonin, 150 mM NaCl, 40 mM HEPES pH 7.4) containing protease inhibitors (1 tablet of complete protease inhibitor cocktail per 10 ml). Cell suspension was gently rotated at 4°C for 5 min. The sample was then centrifuged at 2000 x g. Supernatant which constitutes the cytosol-membrane enriched fraction was recovered and saved. The pellet was resuspended by vortexing in 1 ml of ice-cold buffer 2 (1% NP40, 150 mM NaCl, 40 mM HEPES pH 7.4). Samples were incubated on ice for 30 min and then centrifuged at 7000 x g to pellet nuclei and cell debris. The supernatant which comprises membrane bound organelles such as the ER, Golgi, mitochondria and some nuclear lumenal proteins was recovered and saved. Finally, the pellet was resuspended by vortexing in 1 ml of ice cold buffer 3 (0.5% Na-deoxycholate, 0.1% SDS, 1 U/ml benzonase, 150 mM NaCl, 40 mM HEPES pH7.4). Samples were rotated gently overnight at 4°C to allow complete solubilization of nuclei and digestion of genomic DNA. The supernatant which comprises nuclear membranes and nuclear proteins was finally recovered.

#### Immunofluorescence

HEK 293FT cells grown on glass coverslips and transfected with wild-type or mutant KCC3 were washed in PBS twice at room temperature and fixed with 2% paraformaldehyde in PBS for 30 minutes. The cells were then permeabilized by incubating them twice for 5 min with PBS containing 0.075% saponin (Sigma, St Louis, MO). After blocking with PBS containing 0.075% saponin and 0.2% BSA for 30 minutes, the cells were incubated for 1 h at RT with 150 µl primary antibodies: rabbit polyclonal anti-KCC3 antibody (1∶250, [22]) and mouse monoclonal anti-PDI antibody (Abcam, Ab27043 1∶200), followed by 3×5 min washes with PBS/saponin/BSA and then incubated for 1 h at RT with 150 µl secondary antibodies: Cy3 conjugated anti-rabbit antibody (1∶1000, Jackson Immunochemicals) and Alexa Fluor anti-mouse IgG (H+L) (1∶400, Invitrogen). After final 3×5 min washes in PBS/saponin/BSA solution, coverslips were mounted on slides using VectaShield (Vector Laboratories, Burlingame, CA) and sealed with nail polish. Fluorescence signal was visualized using a Carl Zeiss LMS 510 META confocal microscope.

## Results

**Figure 1:**
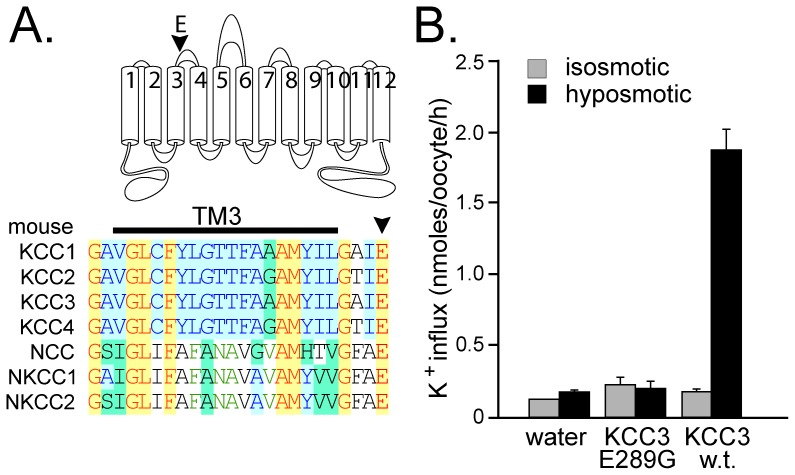
Absence of function of KCC3-E289G mutant cDNA. A) Conservation of glutamic acid residue (E289 in mouse KCC3) within mouse SLC12A cotransporters. The residue, highlighted by an arrowhead in cartoon and sequence alignment, is localized at the end or right downstream of transmembrane domain 3 (TM3). B) K^+^ influx was measured through unidirectional ^86^Rb tracer uptake under isosmotic and hyposmotic conditions, in oocytes injected with water, KCC3-E289G mutant cRNA, and wild-type KCC3 cRNA. Bars represent means±SEM (n = 25 oocytes).

During the process of cloning the mouse KCC3 cDNA, we came across a mutation resulting in the substitution of glutamic acid residue E289 into a glycine (E289G). This negatively charge residue, located at the end of trans-membrane domain 3 (TM3) or beginning of extracellular loop 2 (ECL2) is highly conserved in all mammalian cation-chloride cotransporters (Figure 1A). Although the KCC3-E289G mutant was non-functional when expressed in *Xenopus laevis* oocytes, function was restored when the residue was mutated back into a glutamic acid (Figure 1B).

**Figure 2:**
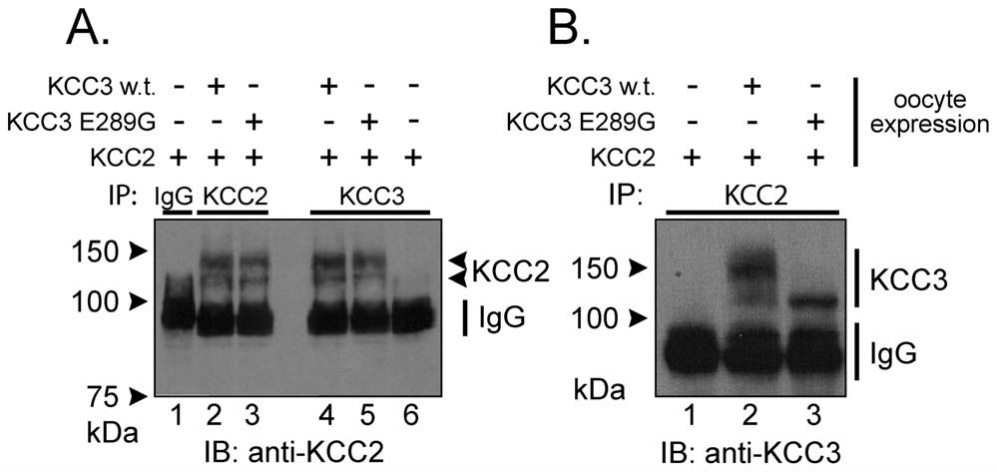
Co-immunoprecipitation reveals interaction between KCC3 and KCC2. *Xenopus laevis* oocytes were injected with KCC2 cRNA in the presence or absence of wild-type KCC3 or KCC3-E289G cRNAs. KCC2 or KCC3 were then immunoprecipitated and the complex was analyzed by Western blot analysis using rabbit polyclonal anti-KCC2 or anti-KCC3 antibodies. Immunodetection of KCC2, KCC3 and IgG are indicated on the right of the panels. Note that both KCC2 (panel A) and KCC3 (panel B) when immunoprecipitated are observed as 2 bands: unglycosylated and glycosylated forms. Experiment was repeated once and yielded similar data.

To demonstrate hetero-dimerization of K–Cl cotransporters, we chose to study the interaction between KCC3 and KCC2, a cotransporter that demonstrates activity under isosmotic conditions. In Figure 2, we co-expressed, wild-type KCC3, and KCC3-E289G in *Xenopus laevis* oocytes and used co-immunoprecipitation to show specific protein-protein interactions. First, we immunoprecipitated KCC3 proteins and immunoblotted for KCC2 (Figure 2, panel A: lanes 4 & 5) or inversely immunoprecipitated KCC2 and immunoblotted for KCC3 (Figure 2, panel B: lanes 2 & 3). No immunoprecipitation was observed with unrelated IgG, and neither antibody cross-reacted with the other K–Cl cotransporter (Figure 2, panel A: lane 6, panel B: lane 1). Lanes 1–3 of panel A constitute internal controls. Note the smaller molecular size of the KCC3-E289G band, indicating possible impairment in glycosylation.

**Figure 3:**
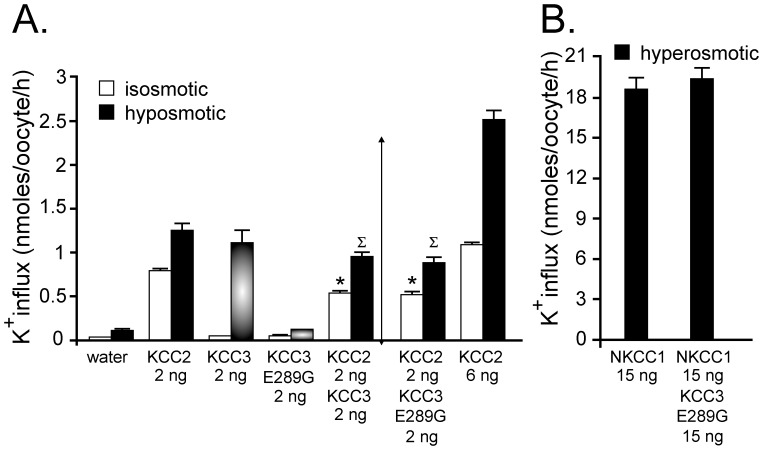
Evidence for dominant-negative effect of KCC3 on KCC2 function. A) *Xenopus laevis* oocytes were injected with KCC2 cRNA in the presence or absence of wild-type KCC3 or KCC3-E289G cRNAs. Three days following injection, K^+^ influx was measured under isosmotic conditions where only KCC2 function is active, or under hyposmotic conditions where both KCC2 and KCC3 function are stimulated. Bars represent mean ± S.E.M. (n = 20–25 oocytes). Flux is expressed in nmoles K^+^/oocyte/h. ^(^*^)^ P<0.01 (ANOVA) compared with KCC2 alone under isosmotic condition, ^(∑)^ P<0.01 (ANOVA) compared with KCC2 alone under hyposmotic solution. Arrow indicates the anticipated flux mediated by the combined activity of KCC2 and KCC3. This is a representative experiment. Each condition (bar) was reproduced multiple times. B) K^+^ influx was measured under hyperosmotic conditions in oocytes injected with NKCC1 cRNA in the presence or absence of KCC3-E289G cRNA. Bars represent mean ± S.E.M. (n = 20–25 oocytes). Flux is also expressed in nmoles K^+^/oocyte/h.

Next, we measured K–Cl transport activity through K^+^ influx measurements in *Xenopus laevis* oocytes (Figure 3A). Under isotonic conditions, KCC2-injected oocytes showed significant K^+^ influx compared to water-injected oocytes. Under isotonic conditions, KCC3-injected oocytes showed no activity, but the activity could be observed upon hyposmotic treatment. When co-injected with wild-type or E289G mutated KCC3, KCC2 activity was significantly reduced compared to KCC2 alone under isotonic conditions. Because KCC3 is inactive under isosmotic conditions, we also examined the effect of KCC3 wild-type and KCC3-E289G on KCC2 under hypotonic conditions. Based on the activity of each cotransporter under hypotonic conditions, we anticipated an additive effect (showed by the arrowed vertical line). However, we again observed a flux that was smaller than KCC2 alone. To demonstrate that this was not due to cRNA saturation, we injected 6 ng KCC2 RNA and observed a significant increase in flux, compared to a 2 ng KCC2 injection. Importantly, we demonstrated that the inhibitory effect of KCC3-E289G on KCC2 function was specific to K–Cl cotransporters, by co-injecting mRNA encoding KCC3-E289G and NKCC1, and observing no inhibitory effect on NKCC1-mediated K^+^ uptake (Figure 3B). This control experiment was important as it demonstrated that the KCC3-E289G mutation did not poison the endoplasmic reticulum.

**Figure 4:**
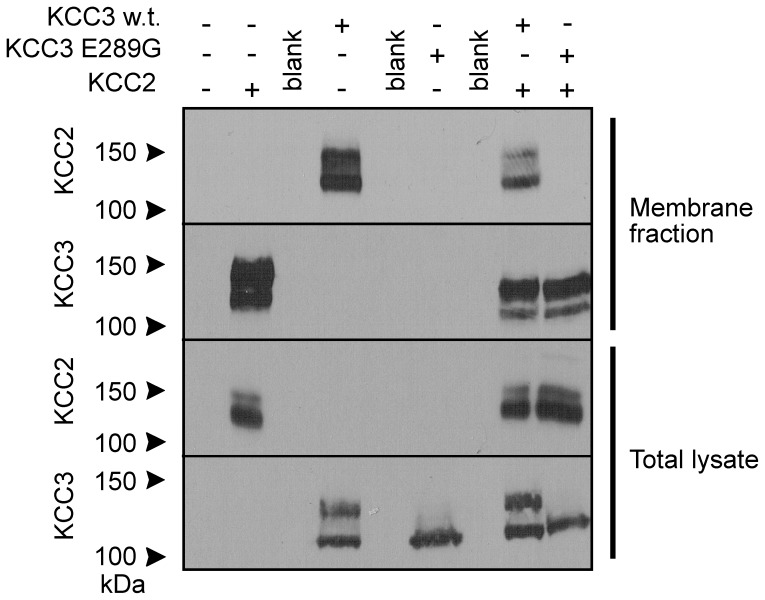
Evidence for dominant-negative effect of KCC3 on KCC2 trafficking. *Xenopus laevis* oocytes were injected with KCC2 in the presence or absence of wild-type KCC3 or KCC3-E289G cRNAs and membrane fractions were isolated using a silica cross-linking method. Membrane proteins and whole oocyte lysates were subjected to Western blot analysis using rabbit polyclonal anti-KCC2 or anti-KCC3 antibodies. Experiment was reproduced 3 times.

**Figure 5:**
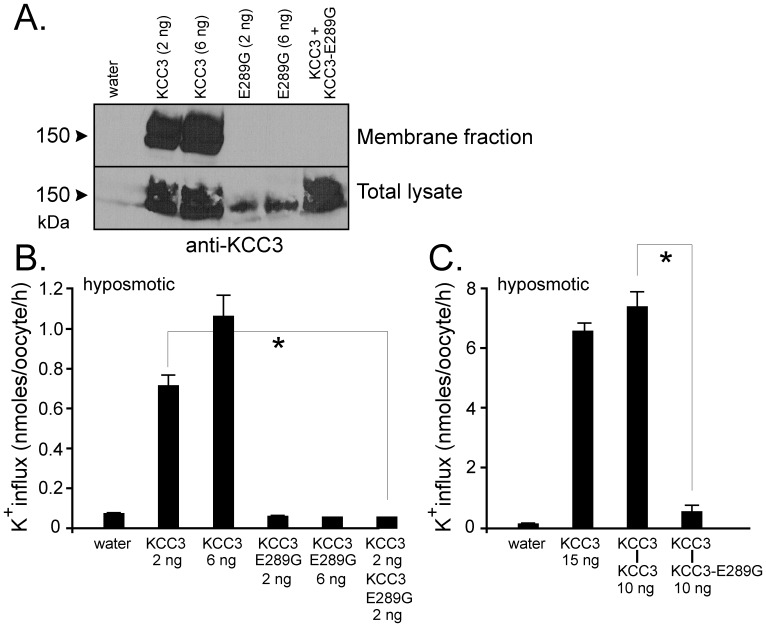
Evidence for dominant-negative effect of KCC3 on KCC2 trafficking and function. *Xenopus laevis* oocytes were injected with KCC3, KCC3-E289G, or both. A) Membrane fraction was isolated using a silica based cross-linking method and membrane proteins and whole oocyte lysates were subjected to Western blot analysis using rabbit polyclonal anti-KCC2 or anti-KCC3 antibodies. B) K^+^ influx was measured under hyposmotic conditions. C) K^+^ influx generated by KCC3-KCC3 and KCC3-KCC3-E289G concatamers. Bars represent mean ± S.E.M. (n = 20–25 oocytes). Flux is expressed in nmoles K^+^/oocyte/h. *P<0.001 (ANOVA) compared with KCC3 controls. Experiment was performed 6 times with similar data.

Using a silica-based cross-linking method [32], we isolated plasma membrane proteins from oocytes injected with KCC2, KCC3, KCC3-E289G, KCC2+KCC3, and KCC2+KCC3-E289G (Figure 4). Consistent with the flux data, we observed absence of KCC3-E289G expression in the plasma membrane and reduced to absent KCC2 expression in the membrane when wild-type KCC3 or KCC3-E289G cRNA was co-injected into oocytes. Similarly, when expressed in oocytes, the KCC3-E289G mutant prevented wild-type KCC3 to reach the plasma membrane (Figure 5A) which resulted in dominant-negative effect on KCC3 function (Figure 5B). As K–Cl cotransporter likely functions as homodimers, we forced dimerization through the use of concatamers or mRNA molecules encoding two KCC3 cotransporters, linked head to tail and separated by a 9-glutamine linker. As seen in Figure 5C, concatamers made of wild-type KCC3 molecules were functional, whereas addition of mutant KCC3-E289G monomer downstream of a wild-type monomer eliminated the function of the dimer.

**Figure 6:**
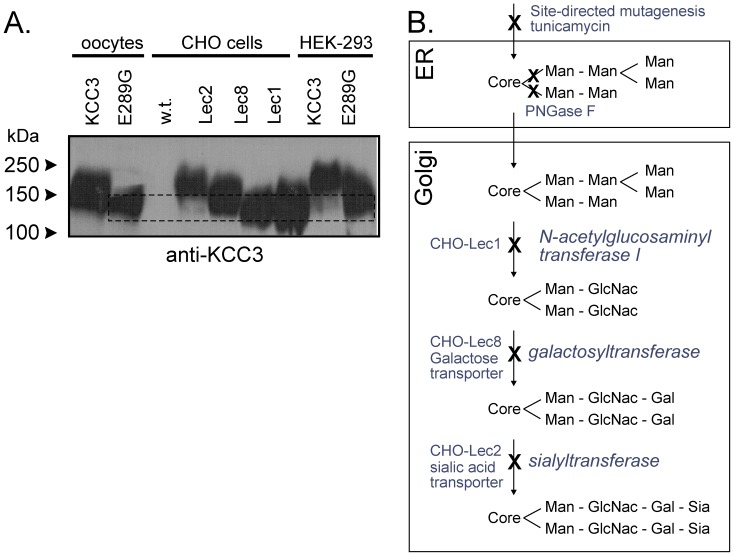
N-Glycolsylation deficiency of the KCC3-E289G mutant. A) Western blot analyses of KCC3-E289G mutant in HEK 293FT cells and *Xenopus laevis* oocytes compared to wild-type KCC3 in HEK 293FT cells, wild-type and mutant CHO cells, and *Xenopus laevis* oocytes, using rabbit polyclonal anti-KCC3 antibody. CHO-Lec1 cells have mutation in N-acetylglucosaminyl transferase, whereas CHO-Lec8 and CHO-Lec2 have deficient galactose and sialic acid transporters, respectively. Two independent experiments are shown. Experiment was performed 4 times. B) Scheme represents the main steps in N-linked oligosaccharide biosynthetic pathway. First, core Glc-Nac-Glc-Nac-Man with branched mannose residues are added to the Asparagines in the ER. In the Golgi, mannose molecules are replaced by acethylglucosamyl groups, followed by the addition of galactose and sialic acid groups. These steps require the availability of galactose and sialic acid in the cells, which is prevented in the mutant CHO cells by elimination of specific transporters.

**Figure 7:**
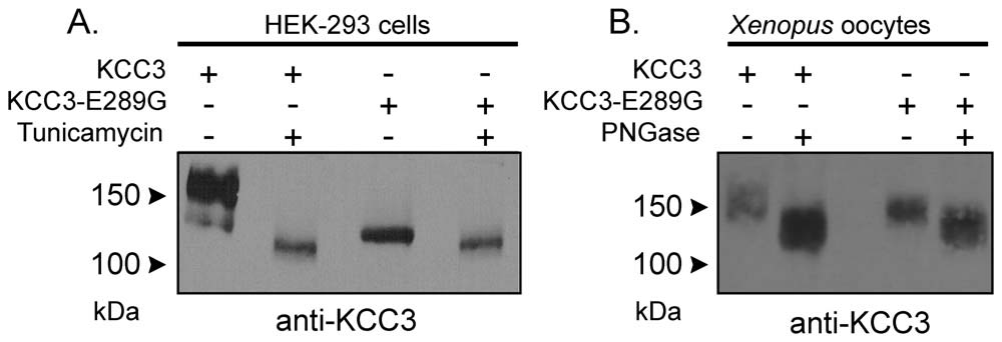
N-Glycolsylation deficiency of the KCC3-E289G mutant. A) Western blot analysis of wild-type KCC3 and KCC3-E289G mutant in HEK 293FT cells treated with tunicamycin (10 µg/ml for 18 h). B) Western blot analysis of wild-type KCC3 and KCC3-E289G mutant proteins isolated from *Xenopus laevis* oocytes and treated with PNGase (0.25U, 12 h at 37°C). The membranes were exposed to a rabbit polyclonal anti-KCC3 antibody. The experiment was repeated once with identical data.

**Figure 8:**
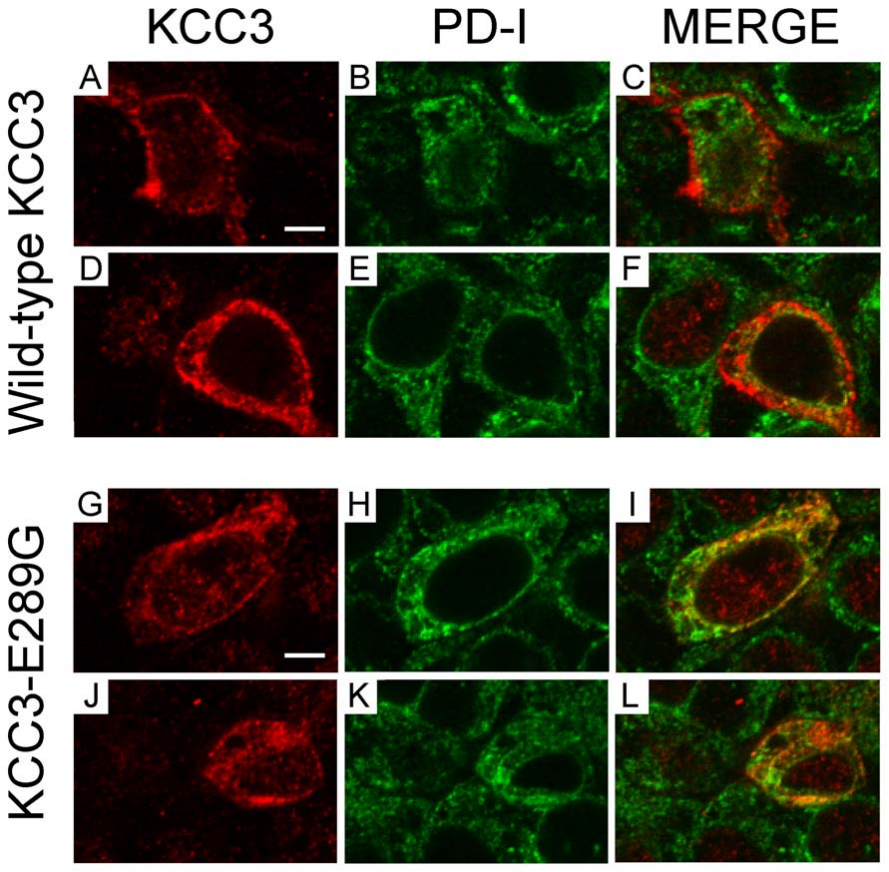
Evidence for KCC3-E289G localizing in the endoplasmic reticulum. HEK 293FT cells were transfected with wild-type KCC3 (A–F) or KCC3-E289G mutant (G–L). Two days post- transfection, the cells were fixed with paraformaldehyde, treated with saponin, and exposed to rabbit polyclonal anti-KCC3 and mouse monoclonal anti-PDI antibodies followed by cy3-conjugated anti-rabbit and Alexa Fluor–conjugated goat anti-mouse antibodies. Focal plane images of KCC3 signal (A, D, J, G), ER marker signal (B, E, H, K), and combined signals (C, F, I, L). Bar = 5 µm.

**Figure 9:**
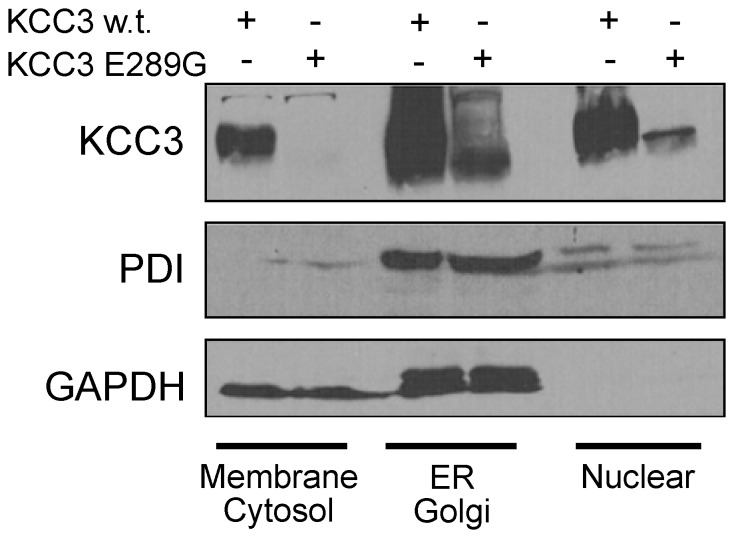
Sub-cellular localization of KCC3 and KCC3-E289G in HEK 293FT cells. HEK 293FT cells were transfected with wild-type KCC3 or KCC3-E289G mutant. Two days post-transfection, the cells were treated with digitonin to extract proteins from cholesterol-rich (plasma) membranes (membrane/cytosol fraction), followed by NP40 treatment to isolate proteins from ER/Golgi fraction, followed by deoxycholate+SDS detergents to isolate proteins from nuclear fraction. Western blots were probed with rabbit polyclonal anti-KCC3 and mouse monoclonal anti-PDI and anti-GAPDH antibodies. Experiment was reproduced at least 5 times with similar data.

The lower molecular size of the KCC3-E289G band observed in Figure 2 indicates a defect in glycosylation. To further substantiate this defect, we transfected HEK 293FT cells with wild-type and mutant KCC3 and observed absence of larger molecular weight products in the KCC3-E289G mutant (Figure 6A). As KCC3 is natively expressed in Chinese hamster ovary (CHO) cells, we took advantage of mutant CHO cell lines (Figure 6B) to evaluate the extent of the KCC3-E289G glycosylation deficit. As shown in Figure 6A, the signal from the KCC3-E289G mutant is similar to the signal shown in the CHO Lec8 and Lec1 samples, indicating early glycosylation deficit. Treatment with tunicamycin, which blocks the synthesis of all N-linked glycoproteins (Figure 7A), and PNGase, an enzyme that cleaves asparagine-linked mannose rich oligosaccharides (Figure 7B), slightly reduced the molecular size of the KCC3-E289G band, indicating the presence of a core glycosylation. These data are consistent with the protein being partially modified in the endoplasmic reticulum. Staining of HEK 293FT cells transfected with wild-type KCC3 (Figure 8A–F) demonstrates cotransporter localization in the plasma membrane and intracellular compartments but with minimal co-localization with the ER marker (PDI). However, the KCC3-E289G (Figure 8G–L) mutant revealed strong co-localization with PDI (Figure 8G–L). Using a subcellular fractionation protocol, we were able to confirm in HEK 293FT cells that the KCC3-E289G mutant does not make it to the plasma membrane, but is exclusively located in intracellular organelles, whereas wild-type KCC3 signal is observed both in membrane and intracellular organelles (Figure 9). No KCC3 signal is observed in water-injected oocytes (over 5 experiments). Note the presence of a sizable fraction of wild-type KCC3 and KCC3-E289G associated in the nuclear fraction, this signal might partially originate from nuclei-associated endoplasmic reticulum, as PDI signal was also observed in the nuclear fraction.

**Figure 10:**
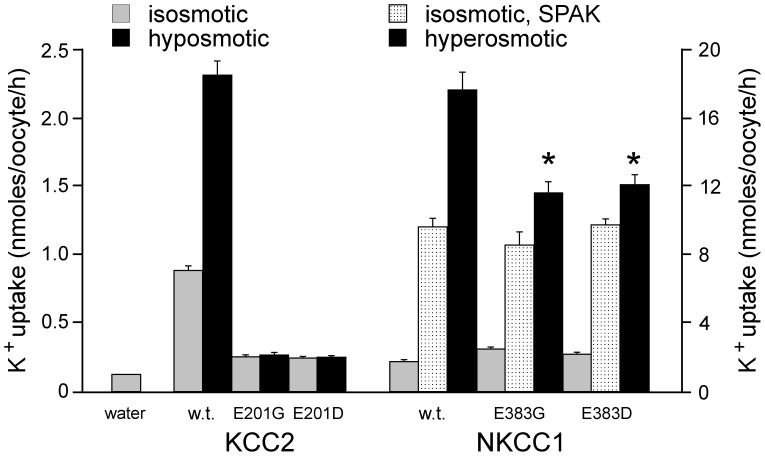
Effect of E289G-like mutations in KCC2 and NKCC1. K^+^ influx was measured through unidirectional ^86^Rb tracer uptake in oocytes injected with water, wild-type KCC2 or KCC2-E201G or KCC2-E201D mutant cRNAs, and wild-type or NKCC1-E383G or NKCC1-E383D mutant cRNAs. Uptakes were measured under isosmotic (200 mOsM) and hypotonic (100 mOsM) solutions for KCC2 and under isotonic and hypertonic (265 mOsM) solutions for NKCC1. Bars represent means±SEM (n = 20–25 oocytes). Fluxes are expressed in nmoles K^+^/oocyte/h. (*) Denotes statistical significance with P<0.001 (ANOVA followed by Tukey-Kramer Multiple Comparisons Test). Experiment was done once.

Because the glutamic acid residue is highly conserved within cation-chloride cotransporters, we examined the effect of mutating the corresponding residues in KCC2 and NKCC1. As seen in Figure 10, mutation of KCC2 glutamic acid residue 201 into glycine completely abrogated KCC2 function under both isosmotic and hyposmotic conditions. Interestingly, the glutamic acid residue could not be substituted with a negatively charged aspartic acid residue. In contrast, there was minimal effect of mutating the corresponding glutamic acid residue in NKCC1, as cotransporter function was similar for mutants NKCC1-E383G or E383D when compared to wild-type NKCC1 under isosmotic conditions. To demonstrate that NKCC1 was expressed in the plasma membrane to similar levels, we utilized a constitutively-active form of SPAK to activate the cotransporter [15], [34]. Under SPAK activation, the levels of cotransporter activity were similar between wild-type and mutant NKCC1cotransporters. However, there was a significant reduction in cotransporter activation in the mutants versus wild-type NKCC1 under hyperosmotic conditions.

## Discussion

K–Cl cotransporters are encoded by four distinct genes each of which generates many variants due to the use of alternative promoters and alternative splicing (For reviews, see [35], [36]). Published Western blot data indicate the presence of dimers or oligomers and functional studies have demonstrated that K–Cl cotransporters form homo-dimers. An intriguing consensus emerging in the field of cation-chloride cotransporter is that cells often co-express multiple isoforms of the K–Cl cotransporter. While this might not necessarily be too surprising for an bipolar epithelial cell that might express one form of the cotransporter on the apical membrane and another on the basolateral membrane, or even for a neuron which might express one form in dendritic spines and another at the cell body, this observation still raises the intriguing possibility that one isoform might affect the expression and/or function of another. To address this possibility, we made use of a functional cotransporter (KCC3), a non-functional cotransporter mutant (KCC3-E289G), and a second functional cotransporter (KCC2) to examine the possibility of hetero-dimerization and co-regulation. The present studies were performed with the specific aim to examine whether a non-functional but full-length cotransporter affects the function of an active cotransporter. We started our studies with a KCC3 mutant that fails to demonstrate hypotonic-stimulation of K^+^ influx when expressed in *Xenopus laevis* oocytes. As the residue is located in an extracellular loop, it is unlikely that the deficit is due to regulation by intracellular signaling/regulatory proteins, such as kinases or phosphatases.

Co-immunoprecipitation experiments revealed that both wild-type and KCC3-E289G mutant interact with KCC2 when co-injected in oocytes. These data indicate that not only KCC2 and KCC3 can be co-expressed in neurons [29], [37], [38], but that they are able to form oligomers. Functional data argue that expression of KCC3 affects KCC2 function or vice-versa, irrespective of the nature of the KCC3 clone co-expressed (wild-type versus mutant). Indeed, it was striking that K^+^ influx measurements were never additive when both cotransporters were expressed in the oocytes. As our experiments were controlled for the level of cRNA injected, it was clear that these effects were true dominant-negative effects and not related to RNA saturation in the oocytes. To explain these data, we examined expression of the cotransporters at the cell surface. Instead of biotinylation, we utilized a method that was developed specifically for *Xenopus laevis* oocytes and which was shown to be of high sensitivity [32]. Protein expressed at the plasma membrane were cross-linked to silica and simply isolated by centrifugation. Indeed, after washing excess silica, lysing the oocytes, pelleting and washing the silica, wild-type KCC2 and KCC3 protein were readily observed by Western blot analysis. In contrast, no signal was observed from oocytes expressing KCC3-E289G, indicating that the mutant cotransporter does not reach the plasma membrane, or if some of it reaches the membrane, it is below the level of detection of the silica method. Co-expression of KCC3-E289G with KCC2 resulted in absence of KCC2 expression in the oocyte membrane, whereas co-expression of wild-type KCC3 with KCC2 resulted in decreased expression of KCC2 in the membrane. These data indicate that the mutant KCC3 protein prevents proper trafficking of KCC2 to the cell surface. The fact that expression of wild-type KCC3 also affects expression of KCC2 is puzzling. This indicates that when the two cotransporters interact, trafficking of this hetero-dimer to the plasma membrane is impaired. However, this interaction would only take place when mRNA molecules are translated simultaneously and the dimer forms in the endoplasmic reticulum. Thus, to prevent this specific mechanism of regulation, cells might have ways to separate translation spatially and/or temporally in order to express full complements of each transporter to the plasma membrane.

Our data indicate that mutation of extracellular glutamic acid residue 289 into a glycine impairs trafficking of KCC3 to the plasma membrane. If trafficking of KCC3-E289G to the cell surface is fully prevented, this by itself explains the absence of function. We attempted to ‘force’ the transporter to the cell surface by using a molecular chaperone utilized to rescue trafficking of the CFTR-Δ508 mutation [39], [40]. Incubation of the oocytes with 2.5 mM 4-phenylbutyrate for 3 days during translation, had no effect on trafficking and function of the mutant KCC3 cotransporter (1002±62 pmoles/oocyte/h (n = 26) for wild-type KCC3 alone versus 1129±42 (n = 26) for wild-type KCC3 in oocytes treated with 4-PBA and 86±12 pmoles/oocyte/h (n = 26) for mutant KCC3 alone versus 90±10 (n = 24) for mutant KCC3 in oocytes treated with 4-PBA). Because the silica method likely has a detection threshold below which there is no cell surface detection, there is a possibility that some of the mutant cotransporter does reach the plasma membrane. If it is the case, this would indicate that the transporters that have reached the membrane are also functionally silent. The precise mechanism by which a single substitution of a glutamic acid residue to a glycine at the end of transmembrane 3 affects trafficking is unknown. It is tempting to speculate that the negatively charged residue at the end of the transmembrane domain affects the threading of the domain across the reticular membrane and that in turn affects the overall topology and folding of the protein. It is, however, important to note that the glutamic acid cannot be substituted by an aspartic acid, which also carries a negative charge (see Figure 10). We did not test additional amino acid substitutions in this study. It will be of interest to expand the analysis to other residues such as glutamine which has a side chain similar to glutamic acid and alanine which has a size similar to glycine. An independent confirmation which supports our observation that the transporter is not properly trafficked to the plasma membrane comes from the migration pattern of the mutant KCC3 protein in an acrylamide gel. Western blot analysis data show absence of a broad band typical of a glycosylated membrane protein for the KCC3-E289G mutant. Treatment of protein lysates isolated from oocytes expressing KCC3-E289 with PNGase demonstrated a small shift in molecular size, indicating that the core glycosylation (or addition of mannose sugars) had taken place in the KCC3-E289G cotransporter mutant. Comparison of the glycosylation pattern of KCC3-E289G in HEK 293FT cells or in *Xenopus laevis* oocytes with the pattern of native KCC3 in CHO cells with mutation in glycosylation genes revealed deficits in the early glycolsylation steps that typically occur in the Golgi. This would indicate that trafficking of the mutant transporter might be arrested in the endoplasmic reticulum or early Golgi. This conclusion was further supported by immunofluorescence data and cell fractionation data that demonstrate co-localization of the KCC3-E289G mutant with the ER marker, PDI. Thus, we have uncovered a glutamic acid residue in KCC3 which when substituted into a glycine affects glycosylation and trafficking of the cotransporter to the plasma membrane. As the mutant cotransporter is full-length it can interact in the ER with wild-type cotransporters (KCC3 or KCC2) and prevent their trafficking as well. Control experiments with co-injection of KCC3-E289G with NKCC1 revealed that trafficking is impaired due to interaction of KCC monomers instead of ER poisoning. As the interaction of K–Cl cotransporters occurs at the C-terminal tail [41], we also tested whether trafficking of KCC2 would be affected by a transporter lacking part of the C-terminal tail. Indeed, co-expression of KCC2 in oocytes with twice the amount of RNA encoding KCC3-T813X, the Quebec HSMN/ACC mutant, had no effect on the function of wild-type KCC2 (2777±162 (n = 25) for KCC2+KCC3-T813X versus 2997±180 (n = 23) for KCC2 alone), indicating that the absence of a C-terminal tail prevents the KCC3-T813X mutant to interact with wild-type and affects its trafficking.

As the KCC3 glutamic acid residue 289 is conserved within cation-chloride cotransporters, we wondered if mutation of the corresponding residue in other K–Cl cotransporters or even one of the Na–K–2Cl cotransporter would also affect their function. Interestingly, KCC2 function was completely eliminated in the KCC2-E201G mutant, whereas function of NKCC1 was only partially affected in the NKCC1-E383G mutant. These data indicate that the residue is far more essential to K–Cl cotransport than Na–K–2Cl cotransporter function. Because the levels of isosmotic transport (under control conditions and SPAK-activated conditions) are identical in the two NKCC1 mutants versus wild-type, it is likely that trafficking of NKCC1 is not affected by the mutations. In contrast, the function of NKCC1 is significantly reduced under hyperosmotic conditions. These data are in agreement with a previous study showing that the second extracellular loop is involved in the sensitivity of NKCC1 and NKCC2 to hyperosmolarity [42].

In conclusion, our results show that E289 in KCC3 and E201 in KCC2 are essential residues for proper trafficking of the cotransporters, respectively. Experiments performed in *Xenopus laevis* oocytes and mammalian HEK 293FT cells revealed that the KCC3-E289G mutant cotransporter is stuck in the endoplasmic reticulum and likely receives only the core mannose glycosylation. Our results also demonstrate that hetero-dimerization of KCC2 and KCC3 is possible and that co-expression of one cotransporter affects expression of the other. These data also indicate that expression of a full-length mutant cotransporter, even if non-functional, might have dominant-negative effects on other related cotransporters, suggesting the possibility that single residue mutations that do not lead to protein truncation might lead to different phenotypes than knockout of the protein.

## References

[1] Gagnon KB, Delpire E (2013) Physiology of SLC12 Transporters: Lessons from Inherited Human Genetic Mutations and Genetically-Engineered Mouse Knockouts. Am J Physiol Cell Physiol In Press. 10.1152/ajpcell.00350.2012 PMC3625803 23325410

[2] Hoffmann EK, Lambert IH, Pedersen SF (2009) Physiology of cell volume regulation in vertebrates. Physiol Rev 89: 193–277. 19126758 10.1152/physrev.00037.2007

[3] Delpire E (2000) Cation-chloride cotransporters in neuronal communication. NIPS 15: 309–312. 11390932 10.1152/physiologyonline.2000.15.6.309

[4] Kahle KT, Staley KJ, Nahed BV, Gamba G, Hebert SC, et al (2008) Roles of the cation-chloride cotransporters in neurological disease. Nat Clin Pract Neurol 4: 490–503. 18769373 10.1038/ncpneuro0883

[5] Blaesse P, Airaksinen MS, Rivera C, Kaila K (2009) Cation-chloride cotransporters and neuronal function. Neuron 61: 820–838. 19323993 10.1016/j.neuron.2009.03.003

[6] Lauf PK, Warwar R, Brown TL, Adragna NC (2006) Regulation of potassium transport in human lens epithelial cells. Exp Eye Res 82: 55–64. 16002066 10.1016/j.exer.2005.05.002

[7] Chen YF, Chou CY, Ellory JC, Shen MR (2010) The emerging role of KCl cotransport in tumor biology. Am J Transl Res 2: 345–355. 20733945 PMC2923859

[8] Jennings ML, Schultz RK (1991) Okadaic acid inhibition of KCl cotransport. Evidence that protein dephosphorylation is necessary for activation of transport by either swelling or N-ethylmaleimide. J Gen Physiol 97: 799–817. 1647439 10.1085/jgp.97.4.799 PMC2216490

[9] Lauf PK, Bauer J, Adragna NC, Fujise H, Zade-Oppen AMM, et al (1992) Erythrocyte K–Cl cotransport: Properties and regulation. Am J Physiol 263: C917–C932. 1443104 10.1152/ajpcell.1992.263.5.C917

[10] Garzón-Muvdi T, Pacheco-Alvarez D, Gagnon KB, Vázquez N, Ponce-Coria J, et al (2007) WNK4 kinase is a negative regulator of K^+^–Cl^−^ cotransporters. Am J Physiol Renal Physiol 292: F1197–F1207. 17182532 10.1152/ajprenal.00335.2006

[11] Kahle KT, Rinehart J, Lifton RP (2010) Phosphoregulation of the Na–K–2Cl and K–Cl cotransporters by the WNK kinases. Biochem Biophys Acta 1802: 1150–1158. 20637866 10.1016/j.bbadis.2010.07.009 PMC3529164

[12] Mercier-Zuber A, O'Shaughnessy KM (2011) Role of SPAK and OSR1 signalling in the regulation of NaCl cotransporters. Curr Opin Nephrol Hypertens 20: 534–540. 21610494 10.1097/MNH.0b013e3283484b06

[13] Pacheco-Alvarez D, Gamba G (2011) WNK3 is a putative chloride-sensing kinase. Cell Physiol Biochem 28: 1123–1134. 22179001 10.1159/000335848

[14] Pacheco-Alvarez D, Vázquez N, Castañeda-Bueno M, de-Los-Heros P, Cortes-González C, et al (2012) WNK3-SPAK interaction is required for the modulation of NCC and other members of the SLC12 family. Cell Physiol Biochem 29: 291–302. 22415098 10.1159/000337610

[15] Gagnon KB, Delpire E (2012) Molecular Physiology of SPAK and OSR1: Two Ste20-Related Protein Kinases Regulating Ion Transport. Physiol Rev 92: 1577–1617. 23073627 10.1152/physrev.00009.2012 PMC4519243

[16] Howard HC, Mount DB, Rochefort D, Byun N, Dupré N, et al (2002) Mutations in the K–Cl cotransporter KCC3 cause a severe peripheral neuropathy associated with agenesis of the corpus callosum. Nat Genet 32: 384–392. 12368912 10.1038/ng1002

[17] Boettger T, Rust MB, Maier H, Seidenbecher T, Schweizer M, et al (2003) Loss of K–Cl co-transporter KCC3 causes deafness, neurodegeneration and reduced seizure threshold. EMBO J 22: 5422–5434. 14532115 10.1093/emboj/cdg519 PMC213773

[18] Labrisseau A, Vanasse M, Brochu P, Jasmin G (1984) The andermann syndrome: agenesis of the corpus callosum associated with mental retardation and progressive sesorimotor neuronopathy. Can J Neurol Sci 11: 257–261. 6329500 10.1017/s0317167100045509

[19] Filteau M-J, Pourcher E, Bouchard RH, Baruch P, Mathieu J, et al (1991) Corpus callosum agenesis and psychosis in Andermann syndrome. Arch Neurol 48: 1275–1280. 1668979 10.1001/archneur.1991.00530240079027

[20] Adragna NC, Chen Y, Delpire E, Lauf PK, Morris M (2004) Hypertension in K–Cl cotransporter-3 knockout mice. Adv Exp Med Biol 559: 379–385. 18727257 10.1007/0-387-23752-6_35

[21] Rust MB, Faulhaber J, Budack MK, Pfeffer C, Maritzen T, et al (2006) Neurogenic mechanisms contribute to hypertension in mice with disruption of the K–Cl cotransporter KCC3. Circ Res 98: 549–556. 16424367 10.1161/01.RES.0000204449.83861.22

[22] Byun N, Delpire E (2007) Axonal and periaxonal swelling precede peripheral neurodegeneration in KCC3 knockout mice. Neurobiol Dis 28: 39–51. 17659877 10.1016/j.nbd.2007.06.014 PMC2242858

[23] Shekarabi M, Moldrich RX, Rasheed S, Salin-Cantegrel A, Laganière J, et al (2012) Loss of Neuronal Potassium/Chloride Cotransporter 3 (KCC3) Is Responsible for the Degenerative Phenotype in a Conditional Mouse Model of Hereditary Motor and Sensory Neuropathy Associated with Agenesis of the Corpus Callosum. J Neurosci 32: 3865–3876. 22423107 10.1523/JNEUROSCI.3679-11.2012 PMC6703451

[24] Salin-Cantegrel A, Riviere JB, Shekarabi M, Rasheed S, Dacal S, et al (2011) Transit defect of potassium-chloride Co-transporter 3 is a major pathogenic mechanism in hereditary motor and sensory neuropathy with agenesis of the corpus callosum. J Biol Chem 286: 28456–28465. 21628467 10.1074/jbc.M111.226894 PMC3151088

[25] Crable SC, Hammond SM, Papes R, Rettig RK, Zhou GP, et al (2005) Multiple isoforms of the KC1 cotransporter are expressed in sickle and normal erythroid cells. Exp Hematol 33: 624–631. 15911086 10.1016/j.exphem.2005.02.006

[26] Gagnon KB, Adragna NC, Fyffe RE, Lauf PK (2007) Characterization of glial cell K–Cl cotransport. Cell Physiol Biochem 20: 121–130. 17595522 10.1159/000104160

[27] Di Fulvio M, Lincoln TM, Lauf PK, Adragna NC (2001) Protein kinase G regulates potassium chloride cotransporter-3 expression in primary cultures of rat vascular smooth muscle cells. J Biol Chem 276: 21046–21052. 11274213 10.1074/jbc.M100901200

[28] Belenky MA, Sollars PJ, Mount DB, Alper SL, Yarom Y, et al (2010) Cell-type specific distribution of chloride transporters in the rat suprachiasmatic nucleus. Neuroscience 165: 1519–1537. 19932740 10.1016/j.neuroscience.2009.11.040 PMC2815043

[29] Pearson M, Lu J, Mount DB, Delpire E (2001) Localization of the K–Cl cotransporter, KCC3, in the central and peripheral nervous systems: expression in choroid plexus, large neurons, and white matter tracts. Neuroscience 103: 483–493. 10.1016/s0306-4522(00)00567-4 11246162

[30] Casula S, Shmukler BE, Wilhelm S, Stuart-Tilley AK, Su W, et al (2001) A dominant negative mutant of the KCC1 K–Cl cotransporter: both N- and C-terminal cytoplasmic domains are required for K–Cl cotransport activity. J Biol Chem 276: 41870–41878. 11551954 10.1074/jbc.M107155200

[31] Delpire E, Gagnon KB, Ledford J, Wallace J (2011) Housing and husbandry of *Xenopus laevis* impact the quality of oocytes for heterologous expression studies. J Am Assoc Lab Anim Sci 50: 46–53. 21333163 PMC3035403

[32] Leduc-Nadeau A, Lahjouji K, Bissonnette P, Lapointe JY, Bichet DG (2007) Elaboration of a novel technique for purification of plasma membranes from *Xenopus laevis* oocytes. Am J Physiol Cell Physiol 292: C1132–C1136. 17079335 10.1152/ajpcell.00136.2006

[33] Holden P, Horton WA (2009) Crude subcellular fractionation of cultured mammalian cell lines. BMC Res Notes 2: 243. 20003239 10.1186/1756-0500-2-243 PMC2802353

[34] Gagnon KB, Rios K, Delpire E (2011) Functional insights into the activation mechanism of Ste20-related kinases. Cell Physiol Biochem 28: 1219–1230. 22179010 10.1159/000335854 PMC7077108

[35] Hebert SC, Mount DB, Gamba G (2004) Molecular physiology of cation-coupled Cl(−) cotransport: the SLC12 family. Pflugers Arch 447: 580–593. 12739168 10.1007/s00424-003-1066-3

[36] Payne JA (2009) The Potassium-Chloride Cotransporters: from Cloning to Structure and Function. In: Alvarez-Leefmans FJ, Delpire E, editors. Physiology and Pathology of Chloride Transporters and Channels in the nervous System: From molecules to diseases. London: Academic Press. pp. 333–356.

[37] Le Rouzic P, Ivanov TR, Stanley PJ, Baudoin FM, Chan F, et al (2006) KCC3 and KCC4 expression in rat adult forebrain. Brain Res 1110: 39–45. 16872584 10.1016/j.brainres.2006.06.055

[38] Shekarabi M, Salin-Cantegrel A, Laganiere J, Gaudet R, Dion P, et al (2011) Cellular Expression of the K(+)–Cl(−) Cotransporter KCC3 in the Central Nervous System of Mouse. Brain Res 1374: 15–26. 21147077 10.1016/j.brainres.2010.12.010

[39] Rubenstein RC, Egan ME, Zeitlin PL (1997) In vitro pharmacologic restoration of CFTR-mediated chloride transport with sodium 4-phenylbutyrate in cystic fibrosis epithelial cells containing delta F508-CFTR. J Clin Invest 100: 2457–2465. 9366560 10.1172/JCI119788 PMC508446

[40] Zeitlin PL, Diener-West M, Rubenstein RC, Boyle MP, Lee CK, et al (2002) Evidence of CFTR function in cystic fibrosis after systemic administration of 4-phenylbutyrate. Mol Ther 6: 119–126. 12095312 10.1006/mthe.2002.0639

[41] Simard CF, Bergeron MJ, Frenette-Cotton R, Carpentier GA, Pelchat ME, et al (2007) Homooligomeric and heterooligomeric associations between K^+^–Cl^−^ cotransporter isoforms and between K^+^–Cl^−^ and Na^+^–K^+^–Cl^−^ cotransporters. J Biol Chem 282: 18083–18093. 17462999 10.1074/jbc.M607811200

[42] Gagnon KB, Delpire E (2010) Molecular determinants of hyperosmotically activated NKCC1-mediated K^+^/K^+^ exchange. J Physiol (Lond) 588: 3385–3396. 20530115 10.1113/jphysiol.2010.191932 PMC2988505

